# Development of a WeChat-based Mobile Messaging Smoking Cessation Intervention for Chinese Immigrant Smokers: Qualitative Interview Study

**DOI:** 10.2196/36091

**Published:** 2022-06-30

**Authors:** Nan Jiang, Erin S Rogers, Paula Cupertino, Xiaoquan Zhao, Francisco Cartujano-Barrera, Joanne Chen Lyu, Lu Hu, Scott E Sherman

**Affiliations:** 1 Department of Population Health Grossman School of Medicine New York University New York, NY United States; 2 Wilmot Cancer Institute University of Rochester Medical Center Rochester, NY United States; 3 Department of Communication College of Humanities and Social Sciences George Mason University Fairfax, VA United States; 4 Center for Tobacco Control Research and Education University of California San Francisco, CA United States; 5 Department of Medicine Veterans Affairs New York Harbor Healthcare System New York, NY United States

**Keywords:** smoking cessation, text messaging, mobile health, Chinese American

## Abstract

**Background:**

Smoking remains a major public health issue among Chinese immigrants. Smoking cessation programs that focus on this population are scarce and have a limited population-level impact due to their low reach. Mobile messaging interventions have the potential to reach large audiences and expand smokers’ access to smoking cessation treatment.

**Objective:**

This study describes the development of a culturally and linguistically appropriate mobile messaging smoking cessation intervention for Chinese immigrant smokers delivered via WeChat, the most frequently used social media platform among Chinese people globally.

**Methods:**

This study had 2 phases. In phase 1, we developed a mobile message library based on social cognitive theory and the US Clinical Practice Guidelines for Treating Tobacco Use and Dependence. We culturally adapted messages from 2 social cognitive theory-based text messaging smoking cessation programs (SmokefreeTXT and Decídetexto). We also developed new messages targeting smokers who were not ready to quit smoking and novel content addressing Chinese immigrant smokers’ barriers to quitting and common misconceptions related to willpower and nicotine replacement therapy. In phase 2, we conducted in-depth interviews with 20 Chinese immigrant smokers (including 7 women) in New York City between July and August 2021. The interviews explored the participants’ smoking and quitting experiences followed by assessment of the text messages. Participants reviewed 17 text messages (6 educational messages, 3 self-efficacy messages, and 8 skill messages) via WeChat and rated to what extent the messages enhanced their motivation to quit, promoted confidence in quitting, and increased awareness about quitting strategies. The interviews sought feedback on poorly rated messages, explored participant preferences for content, length, and format, discussed their concerns with WeChat cessation intervention, and solicited recommendations for frequency and timing of messages.

**Results:**

Overall, participants reported that the messages enhanced their motivation to quit, offered encouragement, and made them more informed about how to quit. Participants particularly liked the messages about the harms of smoking and strategies for quitting. They reported barriers to applying some of the quitting strategies, including coping with stress and staying abstinent at work. Participants expressed strong interest in the WeChat mobile messaging cessation intervention and commented on its potential to expand their access to smoking cessation treatment.

**Conclusions:**

Mobile messages are well accepted by Chinese immigrant smokers. Research is needed to assess the feasibility, acceptability, and efficacy of WeChat mobile messaging smoking cessation interventions for promoting abstinence among Chinese immigrant smokers.

## Introduction

Despite the considerable decline in smoking prevalence in the United States over the past 50 years [1], smoking remains a major public health issue among Chinese American immigrants, particularly men. In New York City, for example, the current (past 30-day) smoking rate among the general male population is 17.5%, whereas the rate for Chinese American men is 28.2% [2]. Foreign-born Chinese Americans are more likely to smoke than those who are born in the United States [2]. The current smoking rate among Chinese American women remains low (1.9%) compared to the general New York City female population (9.2%) [2], reflecting the traditional Chinese cultural norm against women smoking [3].

Culturally and linguistically appropriate smoking cessation programs for Chinese Americans are scarce [4-6]. Moreover, cessation programs targeting this population with demonstrated efficacy, such as the Asian Smokers’ Quitline [7] and community-based cessation programs [8-10], often have limited reach. Utilization of the Asian Smokers’ Quitline is low (1.3%), with an estimated 2010 Chinese-, Korean-, and Vietnamese-speaking smokers using the Asian Smokers’ Quitline every year [11]. This low reach has limited the population impact of the cessation program.

Chinese immigrant smokers face a range of barriers to using smoking cessation programs. These include low awareness of existing programs, skepticism about treatment effectiveness, and time constraints [12-17]. All these factors have deterred Chinese immigrant smokers from using the quitline or local cessation programs. Thus, addressing smoking among Chinese immigrants requires scalable interventions that are engaging, accessible, and efficacious to improve abstinence rates. In addition, Chinese immigrant smokers are largely not ready to quit, with only 6% to 33% planning to quit within a month [18-20]. This is attributed to multiple factors, including low awareness of the harms of smoking and attachment to traditional Chinese norms that support men smoking [12-14,20-23]. Hence, smoking cessation programs targeting Chinese immigrant populations must engage a broad group of smokers, including those who are not ready to quit (ie, those who have no quit date in the next 30 days), in order to optimize the population impact.

Short message service (SMS) cessation programs, which can reach large audiences and expand smokers’ access to treatment, have been shown to be effective in increasing long-term abstinence [24-26]. Current SMS cessation programs, including the National Cancer Institute’s SmokefreeTXT [27] and most clinical trials [26], primarily focus on smokers who are ready to quit (ie, smokers who have a quit date within a month) and exclude unmotivated smokers, who represent a significant portion of smokers. Little is known about the feasibility and treatment efficacy of SMS programs in engaging unmotivated smokers.

To fill this research gap, we are conducting a study to examine the feasibility, acceptability, and preliminary effectiveness of a culturally adapted, linguistically appropriate WeChat-based mobile messaging smoking cessation intervention among Chinese immigrant smokers. WeChat is a social networking application widely used among Chinese people globally [28]. In a pilot study with Chinese immigrant smokers in New York City, we found that WeChat was more frequently used than SMS or other social media sites, such as Facebook, WhatsApp, and Twitter [12]. As part of our larger study, we have developed a theory-based mobile message library tailored to Chinese immigrant smokers. We conducted in-depth interviews to assess content preferences and solicit suggestions to guide further message modifications. In this paper, we present the process of message development and adaptation, including findings from the interviews.

## Methods

Following a guide for developing SMS programs for health behaviors [29], this study consisted of two phases: (1) message development and adaptation and (2) in-depth interviews.

### Phase 1: Development and Adaptation of Mobile Messages

We developed a mobile message library based on social cognitive theory (SCT) and the US Clinical Practice Guidelines for Treating Tobacco Use and Dependence [30]. SCT has been widely used in SMS smoking cessation interventions as a conceptual framework [31-36]. Our messages were designed around key SCT constructs to promote the motivation to quit, increase knowledge and skills related to quitting, improve quitting self-efficacy, and address social norms and misconceptions related to smoking and quitting. Specifically, the content described the adverse health effects of smoking, including light and social smoking, and the harms of exposure to secondhand and thirdhand smoke (ie, behavioral capability); introduced reasons for and benefits of quitting (ie, outcome expectations); encouraged smokers to make quit attempts (ie, self-efficacy); offered advice on quitting preparation (eg, redesigning the environment, knowing smoking triggers, setting a quit date, and securing social support from family and friends) and problem troubleshooting (ie, behavioral capability); provided cognitive and behavioral strategies, such as refusal and coping skills; addressed myths about willpower; described the effect of nicotine replacement therapy; highlighted the prevalence of smoking among Chinese Americans (ie, social norms); and provided information about smoking cessation programs targeting Chinese American smokers.

Our library included messages drawn from 2 SCT-based SMS smoking cessation programs focusing on smokers who are ready to quit, including SmokefreeTXT and Decídetexto (a program designed for Latino smokers [37,38]). We adapted the messages to the cultural context of Chinese immigrant smokers. For example, coping strategies were adapted to include practices relevant to Chinese immigrants (eg, instead of “watching a movie and enjoying a handful of popcorn,” as suggested by the SmokefreeTXT, we suggested the following: “tidy your home/workplace for a few minutes” and “call or text your spouse or friends”).

In addition, we developed new messages targeting smokers not ready to quit in the next 30 days. The content focused on addressing Chinese immigrant smokers’ misconceptions about smoking and quitting (eg, misconceptions related to willpower and nicotine replacement therapy) and common challenges in quitting [12-17,39,40]. New messages were created based on SCT, along with the recommendations of the US Clinical Practice Guidelines for smoking cessation treatment for unmotivated smokers. The new messages included (1) more details about the health hazards of smoking, including light and social smoking (ie, behavioral capability), (2) more motivational and encouraging content (ie, self-efficacy), including passages such as the following: “In your journey of quitting, there may be little stumbles. It’s the progress you are making to achieve success. As long as you get up, it’s not failure. Most former smokers have tried several times before they finally quit. Each quit attempt, even it doesn’t work, you may learn something new about yourself and therefore, you’re a step closer to becoming a former smoker,” (3) more concrete advice on quitting and problem troubleshooting (ie, behavioral capability), (4) emphasis on the smoke-free lifestyle valued by most Chinese Americans (ie, social norms), including information such as the following: “The majority of Chinese immigrants living in New York City do not smoke. This includes 72% of Chinese American men and 98% of Chinese American women,” (5) encouragement to use the Asian Smokers’ Quitline and local language-specific cessation programs (ie, behavioral capability), and (6) myths about willpower and nicotine replacement therapy (ie, behavioral capability).

Messages were developed and adapted through an iterative process. The first author (NJ), a bilingual researcher with English and Chinese proficiency, drafted initial messages in English. Other authors (SES and ESR) reviewed and commented on the messages. Once finalized, all messages were translated into Chinese by the first author and reviewed by another bilingual author (XZ). The messages were not tailored to gender, because for Chinese immigrant smokers, men and women experience similar barriers to cessation (eg, lack of knowledge and quitting skills) and share similar misconceptions (eg, related to willpower) [12].

### Phase 2. In-depth Interviews

#### Participants and Recruitment

Between July and August, 2021, we recruited 20 Chinese immigrant smokers (including men and women) by posting flyers in a community-based organization that primarily serves Chinese Americans and via in-person contacts (ie, snowball sampling). The eligibility criteria included the following: (1) age 18 to 65 years, (2) self-identification as a Chinese immigrant, (3) smoking history of at least 100 total lifetime cigarettes, (4) currently smoking at least 3 days per week, (5) current use of WeChat at least 3 days per week, (6) ownership of a smartphone, (7) ability to speak and read Chinese, (8) residence in New York City, and (9) being somewhat interested in quitting smoking, as assessed by the following screening question: “Which statement best describes your intention to quit smoking?” with response options including “I don’t want to quit at all,” “I may quit at some point, but not in the next 6 months,” “I plan to quit within the next 6 months,” “I plan to quit within the next 30 days,” and “I am currently trying to quit.” Subjects were excluded if they answered “I don’t want to quit at all.” Other exclusion criteria included participation in other smoking cessation services and being pregnant or breastfeeding.

#### In-depth Interview Procedures

Semistructured in-depth interviews were conducted in person. Two research staff moderated each interview in Chinese. Prior to the interview, participants signed written consent, connected with one research staff through WeChat, and completed a brief paper-and-pencil survey of their demographic information, smoking patterns, and WeChat use frequency.

The interviews started with questions about participants’ smoking and quitting experiences and plans for quitting, followed by assessment of the messages. A total of 17 text messages were tested, including 6 educational messages, 3 self-efficacy messages, and 8 skill messages (Table 1). We selected messages that represented various SCT constructs and were relatively long, compared with other messages in the library. The messages were text only, with no videos or pictures. Our research staff sent the 17 text messages to the participants via WeChat during the interview. Participants read each message on their phone and rated them on a rating sheet with a 0 to 10 visual analog scale to indicate to what extent the message enhanced their motivation to quit (for the educational messages), promoted their confidence in quitting (for the self-efficacy messages), and increased their awareness of quitting strategies (for the skill messages). Higher scores indicated higher levels of motivation, confidence, and awareness. The ratings were solely used to facilitate the discussions (eg, to gather feedback on the poorly rated messages), rather than to make comparisons across participants or messages. After the rating was completed, we asked participants what they liked and did not like about the messages, and whether the contents were relevant to them. We gathered feedback on the poorly rated messages to gain insights about content preferences and sought feedback on message length and format. We explored the meanings of key concepts (eg, how participants described substitute behaviors), explored examples of coping and refusal strategies, discussed concerns about using WeChat for smoking cessation intervention, and elicited suggestions for messaging frequency and timing. We also asked participants to summarize each message in order to assess their understanding of the content. The interviews typically lasted between 60 and 90 minutes. The interviews were audio-recorded and transcribed into Chinese. Participants were compensated with a US $75 gift card.

**Table 1:** Text messages assessed in the interview.

Content of messages			Social cognitive theory construct		Number of messages (total=17)
**Educational messages**					
	Harms of smoking	Behavioral capability		2	
	Health benefits of quitting	Outcome expectations		1	
	Reasons for quitting	Outcome expectations		1	
	Key elements in quitting	Behavioral capability		1	
	Social and light smoking	Behavioral capability		1	
Self-efficacy messages			Self-efficacy		3
**Quitting skill messages**					
	Redesign environment	Behavioral capability		1	
	Identify smoking triggers	Behavioral capability		1	
	Explore substitute behaviors to cope with cravings	Behavioral capability		2	
	Refusal strategies	Behavioral capability		1	
	Secure social support from family and friends	Behavioral capability		1	
	Set a quit date	Behavioral capability		1	
	Misconception: willpower	Behavioral capability		1	

#### Data Analysis

The data were analyzed with NVivo version 12 (QSR International). The first author closely read all transcripts (in Chinese) and created an initial codebook of themes and subthemes in 3 domains related to the research questions (eg, quitting experience, perceptions about the messages, and attitudes toward the WeChat mobile messaging smoking cessation intervention). Two coders (NJ and another bilingual team member) independently coded a subset of transcripts, generated emergent themes and subthemes using an inductive analytic approach [41], discussed and resolved disagreements on the codes, and finalized the codebook. The first author then coded the remaining transcripts and selected illustrative quotes. The quotes were reviewed by both coders and translated into English.

### Ethics Approval

The study protocol was approved by the Institutional Review Board of New York University Grossman School of Medicine (i20-01959).

## Results

Participants (age range 24-62 years) included 7 women, 13 daily smokers, and 7 nondaily smokers (Table 2). Thirteen participants were cigarette-only smokers and 7 reported dual use of cigarettes and e-cigarettes. Participants reported an average of 7.3 (SD 3.3) years of residence in the United States. Half of our participants were restaurant staff. A total of 9 main themes and 5 subthemes emerged from the data.

**Table 2:** Demographic and smoking characteristics of participants (N=20).

Characteristics		Values
**Gender, n (%)**		
	Male	13 (65)
	Female	7 (35)
Age, mean (SD) years		37.4 (10.4)
Length of residence in the United States, mean (SD) years		7.3 (3.3)
**Education, n (%)**		
	Middle school or less	4 (20)
	High school or vocational high school	10 (50)
	Some college, no degree or associate degree	4 (20)
	Bachelor’s or advanced degree	1 (5)
	Unreported	1 (5)
**Occupation, n (%)**		
	Restaurant staff	10 (50)
	Hairdresser	5 (25)
	Taxi driver	2 (10)
	Housewife	2 (10)
	Retired	1 (5)
**Current smoking status, n (%)**		
	Nondaily smoker	7 (35)
	Daily smoker	13 (65)
Cigarette consumption per day, mean (SD)		9.4 (5.4)
Age of smoking initiation, mean (SD) years		19.8 (5.9)
**Current e-cigarette use, n (%)**		
	Yes	7 (35)
	No	13 (65)

### Domain 1: Quitting Experience

#### Theme 1: Quit Attempt and Intention

Nineteen participants reported that they had tried to quit, but only 2 had used evidence-based quitting methods (both had used nicotine patches or gum). Fourteen participants wanted to quit completely, and their reasons were related to concerns for their own health and the health of their children. Of the 14 participants, 4 planned to quit immediately or after finishing the last few packs of cigarettes, 1 planned to quit in 3 to 4 months, 2 stated that they would quit in the future, when they planned to have a baby, and 7 were interested in quitting but had no plans about when to quit.

I want to quit completely, including cigarettes and e-cigarettes, because smoking is bad for health. It hurts my lungs. But I haven’t thought about when to quit. Probably in the future when something happens to me, I will make the determination to quit.

Participant #4, male, 27 years old, daily smoker

#### Subtheme: Reasons for Lacking Determination in Quitting

Participants who were ambivalent about quitting discussed why they were not determined to quit. Restaurant staff often claimed that smoking was their only excuse for taking a break from work, which had a big impact on their decision. Other factors included perceived low risks of smoking, low confidence in quitting, and need for socializing with friends.

I don’t see the possibility to quit because my job [as restaurant staff] requires high-intensity labor work. We’re busy all the time and I’m exhausted. We have no breaks because the boss doesn’t allow us to take a break. I have the excuse because I smoke so I can take a short break. If I quit, I would no longer have an excuse. So I’m not gonna quit unless I change the job.

Participant #16, male, 37 years old, daily smoker

I don’t think I need to quit, because smoking doesn’t seem to have an effect on me. It may because I don’t smoke much... I have a minor issue with breathing but I’m not sure if it’s related to smoking.

Participant #14, female, 36 years old, nondaily smoker

I don’t want to quit because I tried [to quit] last time and I couldn’t deal with the withdrawal. It was miserable.

Participant #1, male, 29 years old, daily smoker

### Domain 2: Perceptions About the Messages

#### Theme 2: Likes About Educational Messages

More than half of the participants reported that the messages provided them with information that they were unaware of before, particularly about the adverse health effects of smoking. Some participants were in favor of the content about how smoking damages the body.

Cigarettes contain lots of toxic chemicals which is bad for health. I know it. But I don’t know the exact harms caused by smoking or how exactly it damages the body. The messages provide detailed, comprehensive information about the harms, and explain clearly how smoking affects health… I know smoking causes lung cancer, but I don’t know it causes stroke and heart disease as well. This is new [knowledge] to me.

Participant #15, male, 39 years old, daily smoker

#### Subtheme: Change in Motivation to Quit

Half of the participants reported that, after reading the messages, they would consider quitting (among those who were ambivalent about quitting) or became more motivated to quit (among those who planned to quit). This could be in part because they became more informed about the health impacts of smoking.

The messages about the dangers of smoking really scared me! While reading the messages, I was thinking “oh no, my heart cannot go wrong. I’m still young.” I think quitting is important.

Participant #8, female, 36 years old, daily smoker

#### Subtheme: Factors That Influenced Participant Decisions to Quit

One message described common reasons why smokers want to quit. When asked to select factors that were important in their decision-making about quitting, participants generally chose health-related items (eg, “be healthier,” “look healthier,” “improve the health of people around you,” and “enjoy better sexual and reproductive health”). Participants rarely selected other items (eg, “save money,” “live in a better environment,” “live longer,” “be free of addiction,” and “shape your family and community”). When asked why saving money was not among the top factors that influenced their decisions, nondaily and light smokers noted that the cost of smoking was only a small portion of their daily expenses. Heavy smokers often reported that they had access to cheaper cigarettes through alternative sources.

I smoke a pack every 2 or 3 days so it’s like $3 per day. This is a tiny amount of money, just like a cup of coffee or a piece of bread... People do not spend hundreds of dollars to buy cartons of cigarettes at one time. So no one will make the calculation to see how much it [smoking] would cost for a day, for a month, and for a year.

Participant #10, male, 28 years old, daily smoker

#### Theme 3: Likes About Self-Efficacy Messages

Many participants reported that the self-efficacy messages offered encouragement. They particularly favored the content emphasizing the role of the environment and quitting skills in successful attempts. Participants expressed strong agreement with the message emphasizing that quitting takes many attempts.

The messages pointed out the importance of environment, which is THE reason why I failed years ago. It tells exactly what I feel… While reading these messages, I felt more confident in myself.

Participant #19, female, 51 years old, daily smoker

For the majority of smokers, quitting is impossible to achieve with one or two tries. “There is no real failure in the process of quitting.” This is a great point. For those who want to quit, knowing this will make them more confident in themselves.

Participant #10, male, 28 years old, daily smoker

#### Theme 4: Dislikes About Self-Efficacy Messages

According to some participants, content about the influence of a smoker’s success in quitting on his or her community was exaggerated. Participants felt that quitting is “not a big deal” (Participant #17, female, 36 years old, nondaily smoker).

I don’t think other people really care whether you quit or not. If you quit, you may feel good about yourself. You may think you’re successful. But it’s not a big achievement. It’s your own business. It cannot inspire others.

Participant #15, male, 39 years old, daily smoker

#### Theme 5: Likes About Skill Messages

Participants overwhelmingly favored the skill messages and described them as “useful,” “concrete,” “comprehensive,” and “relevant.” Most quitting strategies were perceived to be feasible, including redesigning the environment, identifying smoking triggers, and using substitute behaviors to cope with nicotine cravings. Participants claimed they were willing to apply the strategies in future quit attempts. When exploring appropriate ways to refuse offered cigarettes, participants preferred to say “no” directly, claim that they have quit, or give a reason or an excuse (eg, “I plan to have a baby” or “my throat hurts”).

The suggestions are very concrete. … The information is comprehensive, complete, and perfect! As long as you follow the guidance, you will definitely succeed!

Participant #6, male, 56 years old, daily smoker

#### Theme 6: Mixed Opinions About Skill Messages

Participants reported mixed opinions toward engaging with their social support networks in the quitting process. Female smokers were generally in favor of social support, and they had all applied this strategy in previous quit attempts. In contrast, male smokers were often reluctant to engage family or friends. Some stated that “quitting is a personal decision.” Some noted that family’s criticism about their quitting efforts undermined their determination, which explained their preference against engaging social networks in quit attempts.

If I decide to quit, I’ll tell my family and friends. I’ll ask them to remind me. I think it will be helpful.

Participant #14, female, 36 years old, nondaily smoker

I will not purposively tell others that I’m quitting. It’s your own business.

Participant #13, male, 29 years old, daily smoker

[If I’m going to quit] I won’t tell my family. Dealing with cravings is already very hard. They [my family] are likely to keep nagging and making sarcastic comments [on my slip] and the thing [quitting] will eventually go the wrong way...

Participant #2, male, 30 years old, daily smoker

Participants also had different opinions about setting a quit date. Half of the participants favored using Mondays as a quit (or requit) date because (1) “it makes me more want to try [quitting] because even I fail I can always do it again” (Participant #14, female, 36 years old, nondaily smoker), (2) “The date is fixed. It’s easy to remember” (Participant #9, male, 62 years old, daily smoker), and (3) “I can see my progress... The first week I may stay abstinent [from Monday] till Tuesday. Next week I may stay abstinent till Wednesday” (Participant #15, male, 39 years old, daily smoker). Other participants were skeptical about the effect of setting a quit date or preferred to have their own quit date.

No one can really quit from the quit date so there’s no need to set a date. If you decide to quit, do it now!

Participant #5, male, 27 years old, daily smoker

#### Subtheme: Challenges in Applying the Quitting Strategies

Participants, particularly restaurant staff, discussed challenges in redesigning the environment. They reported that it was nearly impossible to create a supportive environment because most coworkers smoked. Participants also noted the challenge of using substitute behaviors to cope with craving and stress. For taxi drivers and restaurant staff, their stress from work was often a trigger to smoke, but they did not know how to deal with it.

I work in the kitchen. All the cooks smoke, so it’s impossible to have a good environment to quit.

Participant #19, female, 51 years old, daily smoker

I only smoke during the working days. The kitchen work is very busy. … Every few hours, I tell the manager that “I need to smoke.” So I can have a break and relax for a few minutes.

Participant #17, female, 36 years old, nondaily smoker

#### Theme 7: Readability, Format, Length, and Language Clarity

Participants were generally able to summarize the messages appropriately and captured the key content. They expressed a preference for messages written in bulleted lists and limited to approximately 380 Chinese characters (including spaces). That length fits within about a single screen of a mobile phone (in the default font). Participants were reluctant to read long paragraphs and some would skip those long messages.

Participants were not familiar with some terms, including “quit date,” “social smoking,” and “substitute behavior.” How we translated the terms into Chinese influenced their understanding of the concepts.

I’ve never heard of “quit date.” Does it mean you cannot start quitting until the “quit date”?

Participant #13, male, 29 years old, daily smoker

### Domain 3: Attitudes Toward the WeChat Mobile Messaging Smoking Cessation Intervention

#### Theme 8: Attitudes Toward the WeChat Cessation Intervention

Participants expressed strong interest in the WeChat smoking cessation intervention. They felt that the intervention would provide them with useful information, take a minimum of their time, and not require them to quit at a certain date or take medications.

I’d like to participate because it [the intervention] does not force me to do anything, like no smoking starting from a certain day... It’s like a daily reminder. It may help me reduce smoking.

Participant #1, male, 29 years old, daily smoker

#### Subtheme: Privacy Concerns

The majority of participants reported no concerns about joining the WeChat mobile messaging cessation intervention, except 2 smokers who noted concerns about privacy issues associated with using WeChat.

#### Theme 9: Suggestions on the Frequency and Timing of Messages

All the participants reported that receiving 1 daily message was acceptable. Some noted that “2 or 3 messages per day” or “no more than 5 messages per day” would be appropriate. Preferences for the timing of the messages varied, ranging from morning (“8:00 AM when I go to work”) to late night (“10:00 PM when I finish a whole day work”). Some participants had no preference for timing, because they had no or minimal access to their mobile phone while at work or they checked messages only when they wanted to.

I usually check [WeChat] messages when I wake up in the morning and after work… While at work, we put phones away at the cashier. We get phones back after work or if we need to make an emergent call.

Participant #17, female, 36 years old, nondaily smoker

## Discussion

### Principal Findings

This study describes the development of a WeChat-based mobile messaging program for smoking cessation tailored to Chinese immigrant smokers. Our findings provide important insights into how smokers perceived the messages and their attitudes toward the WeChat mobile messaging cessation intervention. In general, the messages were well-received. Participants described the messages as useful and relevant, and claimed that the content enhanced their motivation to quit and offered encouragement. Participants particularly liked messages about the harms of smoking and strategies for quitting (eg, coping skills), and reported that they learned new information from these messages. This finding affirms the low health literacy for smoking and lack of quitting skills among Chinese immigrant smokers. Previous studies have reported that Chinese immigrant smokers are often unclear about the exact health effects of smoking, and that many smokers inaccurately believe that quitting would result in health problems or that willpower is the only key to cessation [12,13,15-17,40]. Our findings indicate that smoking cessation programs that work with Chinese immigrants have to include concrete information about the specific dangers of tobacco use, the health benefits of quitting, and detailed guidance on how to quit.

A noteworthy finding is that the factor that most impacted participant decision-making on quitting was concern for their health and the health of their children. Other factors, such as economic gains, did not seem to have an equal effect in motivating smokers to quit. Among smokers who planned to quit, all cited health concerns as the reason for quitting. Findings suggest that cessation programs for Chinese immigrant smokers need to emphasize the health impacts of smoking, rather than stress the financial impacts.

Participants expressed a strong interest in the WeChat mobile messaging cessation intervention for several reasons. First, they felt the messages were helpful. Second, they thought that the intervention would take a minimum of time. Thus, they perceived no barriers to engagement. First-generation immigrants often report long and inflexible working hours, which prevents them from participating in quitlines or in-person cessation treatment programs [12-14,17]. Mobile messaging intervention has the potential to accommodate immigrant smokers, because they can access messages at their own pace and at the time of their convenience. Leveraging the WeChat platform made the cessation intervention more accessible. Third, unlike most SMS cessation programs that solely focus on smokers who are committed to quitting (with a self-determined quit date in the next 30 days) [25-27], our intervention was designed to target a larger audience, including smokers not ready to quit. This appealed to our participants, who claimed that they would give it a try because the intervention “does not force me to quit.” Our next step involves testing the feasibility, acceptability, and effectiveness of the WeChat mobile messaging cessation intervention among Chinese immigrant smokers.

We identified needs for message modifications. For example, our message library should include more content about how to cope with stress and offer tips on how to stay abstinent at work. Several studies have reported that stress is a primary trigger for smoking among Chinese immigrant smokers and that the lack of coping skills is an important factor associated with the low intention to quit [12,39]. In addition, smoking is more prevalent among workers in certain occupations (eg, food preparation and services) [42]. Smokers working in these industries may encounter more difficulties in quitting. Hence, an emphasis on capacity building, including how to manage stress and deal with workplace smoking, should be integrated into smoking cessation programs that focus on immigrant smokers. We also identified a preference for the message format used in our WeChat mobile messaging intervention. Paragraphs should be written in bulleted lists when possible. Long paragraphs are not appropriate, since they are hard to read on mobile phones.

### Limitations

The interviews were conducted in one geographic area with Chinese immigrant smokers who were already somewhat interested in quitting. The findings may not be generalizable to other areas or to smokers who are not at all interested in quitting. Moreover, the interviews were all conducted in Mandarin. We lack data and perspectives on the messages and attitudes toward the WeChat mobile messaging cessation intervention from smokers who speak Cantonese or other dialects. However, Chinese immigrants in New York City are mostly able to communicate in Mandarin, even if they primarily speak Cantonese [43].

### Conclusions

This study contributes to the small body of literature on the development process of mobile messaging smoking cessation interventions tailored to specific racial or ethnic minority groups. The participants in our study claimed that the messages enhanced their motivation to quit and offered encouragement. Their report on barriers to quitting guided us in modifying the messages and generating new ones. The participants were strongly interested in the WeChat mobile messaging cessation intervention and noted its potential to address their access barriers to existing smoking cessation programs. We will follow up on this qualitative study with feasibility testing and a randomized controlled trial to explore if a WeChat mobile messaging cessation intervention is acceptable, and to what extent it can help Chinese immigrant smokers achieve abstinence.

## Abbreviations

SCT: social cognitive theory
SMS: short message service
